# Impact of Type 2 Diabetes Mellitus on Endometrial Cancer Survival: A Prospective Database Analysis

**DOI:** 10.3389/fonc.2022.899262

**Published:** 2022-05-05

**Authors:** Kelechi Njoku, Heather J. Agnew, Emma J. Crosbie

**Affiliations:** ^1^Division of Cancer Sciences, School of Medical Sciences, Faculty of Biology, Medicine and Health, St Mary’s Hospital, University of Manchester, Manchester, United Kingdom; ^2^Stoller Biomarker Discovery Centre, Institute of Cancer Sciences, Faculty of Biology, Medicine and Health, University of Manchester, Manchester, United Kingdom; ^3^Department of Obstetrics and Gynaecology, Manchester University NHS Foundation Trust, Manchester Academic Health Science Centre, Manchester, United Kingdom

**Keywords:** endometrial cancer, prognosis, survival, type 2 diabetes mellitus, mortality

## Abstract

**Purpose:**

Type 2 diabetes mellitus (T2DM) is an established risk factor for endometrial cancer but its impact on endometrial cancer survival outcomes is unclear. The aim of this study was to investigate whether pre-existing T2DM impacts survival outcomes in endometrial cancer.

**Patients and Methods:**

Women diagnosed with endometrial cancer were recruited to a single centre prospective cohort study. Relevant sociodemographic and clinico-pathological data were recorded at baseline. T2DM status was based on clinical and biochemical assessment, verified by general practitioner records and analysed in relation to overall, cancer-specific and recurrence-free survival using Kaplan-Meier estimation and multivariable Cox-regression.

**Results:**

In total, 533 women with median age and BMI of 66 years (Interquartile range (IQR), 56, 73) and 32kg/m^2^ (IQR 26, 39) respectively, were included in the analysis. The majority had low-grade (67.3%), early-stage (85.1% stage I/II), endometrial cancer of endometrioid histological phenotype (74.7%). A total of 107 (20.1%) had pre-existing T2DM. Women with T2DM had a two-fold increase in overall mortality (adjusted HR 2.07, 95%CI 1.21-3.55, p=0.008), cancer-specific mortality (adjusted HR 2.15, 95% CI 1.05-4.39, p=0.035) and recurrence rates (adjusted HR 2.22, 95% CI 1.08-4.56, p=0.030), compared to those without, in multivariable analyses.

**Conclusion:**

T2DM confers an increased risk of death in endometrial cancer patients. Well-designed longitudinal studies with large sample sizes are now needed to confirm these findings.

## Introduction

Endometrial cancer is the sixth most common cancer in women globally and the most common gynaecological malignancy in high-income countries. Worldwide, there were an estimated 417,000 incident cases and 97,000 deaths in 2020 (1). The incidence of endometrial cancer is rising year on year, in line with the obesity epidemic (2). Deaths from endometrial cancer are also rising, albeit at a slower rate, despite improvements in overall survival (3, 4). Although most women with endometrial cancer are diagnosed with highly curable disease and have a favourable prognosis, a significant minority present with adverse clinico-pathological characteristics that portend poor outcomes (5).

Identifying women with endometrial cancer who are at a higher risk of relapse and cancer-related mortality is fundamental to ensuring women receive appropriate evidence-based management whilst minimising the side effects and costs of unnecessary interventions for those at lowest risk (6). In current clinical practice, endometrial cancer risk assessment is based on clinico-pathological parameters including International Federation of Gynecology and Obstetrics (FIGO) surgical stage, tumour grade and histological subtype, lymphovascular space invasion and depth of myometrial invasion. The molecular classification of endometrial cancer offers a more objective and reproducible endometrial cancer risk assessment compared with traditional histopathological evaluation (7, 8). Age, body mass index (BMI) and comorbidity status are other predictors of outcomes that are often taken into consideration in treatment algorithms (9). A retrospective analysis of 671 patients with FIGO stage I-II endometrioid endometrial cancer found that higher age-adjusted comorbidity scores are associated with worse outcomes (10). Indeed, cardiovascular events are the leading cause of death amongst endometrial cancer survivors (11).

Type 2 diabetes mellitus (T2DM) is an important risk factor and a common comorbidity in women with endometrial cancer (12). A meta-analysis of 13 primary studies adjusting for BMI concluded that women with T2DM have a 62% increase in the risk of endometrial cancer, independent of obesity (13). Mechanistically, insulin resistance and the resultant hyperinsulinemia promotes endometrial carcinogenesis and progression by the direct pro-proliferative and anti-apoptotic effect of insulin and insulin growth factor (IGF-1) on endometrial cells (14, 15). Whether T2DM also impacts on outcomes following diagnosis and treatment for endometrial cancer is unclear. The meta-analysis of six prospective cohort studies by Zhang and colleagues reported that there was insufficient evidence for an association between T2DM status and endometrial cancer mortality (16). A more recent meta-analysis of five cohort studies by Liao and colleagues concluded that the data linking T2DM and endometrial cancer-specific mortality are inconsistent and the association uncertain (17).

The aim of this study was to investigate whether pre-existing T2DM impacts on survival outcomes in endometrial cancer patients in a large prospective database study.

## Methods

### Study Population

Women with a diagnosis of endometrial cancer who were treated between 2010 and 2016 at St Mary’s Hospital, a regional specialist centre for the management of gynaecological malignancies, were eligible for inclusion. All study participants gave written informed consent for their pseudo-anonymised data to be used for future research. We collected relevant sociodemographic and clinico-pathological data, including age, BMI, T2DM status, socioeconomic quintile, histological subtype, tumour grade and stage, depth of myometrial invasion, lymphovascular space invasion (LVSI) and baseline serum C-reactive protein (CRP). Age at diagnosis was dichotomised into <65 and ≥65 years, consistent with previous studies, and women were classed as underweight (BMI<18.5 kg/m^2^), normal weight (BMI 18.5-24.9kg/m^2^), overweight (BMI 25-29.9kg/m^2^) or obese (BMI≥30kg/m^2^) in line with the World Health Organisation BMI groupings. Endometrial cancers were classified according to histological subtype (endometrioid, serous, clear cell, carcinosarcoma) based on expert histopathology review by two specialist gynaecological pathologists, reporting according to the UK Royal College of Pathology standards and using FIGO 2009 surgical staging classification.

The primary treatment for most women was surgical with total hysterectomy and bilateral salpingo‐oophorectomy. Women with intermediate and high-risk disease were offered adjuvant therapy in accordance with national and international guidelines (9, 18). A small minority of women with grade 1 stage IA endometrial cancer who wished to preserve their fertility, or who were medically unfit for surgery, were managed conservatively with primary hormonal therapy (+/-delayed hysterectomy). A few women received primary palliative radiotherapy.

All cases were reviewed in follow-up clinics at 3‐month (for 3 years), 6‐month (for 1 year) and 12‐month intervals for a total duration of 5 years, or until relapse or death, whichever was sooner. Where women had completed routine hospital-based follow up or moved away from Manchester, general practitioners were contacted to ascertain their current status. Women who relapsed during follow up were managed according to national and international guidelines (9, 18). Those with local pelvic recurrence were treated surgically or with radiotherapy as appropriate, whereas those with wide-spread metastatic or distant recurrent disease were managed with palliative hormone therapy, chemotherapy +/- radiotherapy. The cause of death was based on information obtained from death certificates.

### Statistical Analysis

The study end-points were overall, cancer-specific and recurrence free survival. Overall survival was calculated from primary treatment initiation to death from any cause or the last day of availability of survival data. Cancer‐specific survival was calculated from initiation of primary treatment to death from endometrial cancer or the date of last follow-up, and censored on date of death from other causes. Recurrence‐free survival was calculated from primary treatment initiation to the first record of disease recurrence, death or date of last follow-up, whichever was sooner. Chi-square (X^2^) and Fisher’s exact tests were used to test for associations between categorical variables, as appropriate. Student’s t-test and one-way or two-way ANOVA was used to test for statistical significance for continuous variables as indicated. We used the Kaplan–Meier method to compute survival rates and the log‐rank test was used to assess survival differences between groups. Cox regression multivariable modelling was used to evaluate the association between T2DM status and the study end-points while adjusting for confounding and effect modifications. We computed hazard ratios (HRs) with 95% confidence intervals (95% CIs) for both univariable and multivariable analyses. The confounding variables adjusted for in the models were age at diagnosis, BMI, FIGO stage, histological subtype, grade, LVSI, depth of myometrial invasion, socioeconomic quintile and baseline CRP. We assessed for confounding by evaluating the changes in hazard coefficients following the introduction of these variables to the Cox regression models. We assessed for the assumptions of proportional hazards which was met for all models. A p-value of <0.05 was considered statistically significant. All analyses were conducted using the statistical package Stata 16.0 (https://www.stata.com).

## Results

### Descriptive Characteristics of the Study Population

In total, 533 women with histologically confirmed endometrial cancer were included in this analysis (**Table 1**). Their median age and BMI were 66 years (Interquartile range (IQR), 56, 73) and 32kg/m^2^ (IQR 26, 39) respectively. Most women were overweight or obese (83.5%) and aged ≥65 years (54.4%). One-fifth of the study population (20.1%) had pre-existing T2DM. The modal socioeconomic quintile was quintile I (most deprived) and accounted for 37.0% of the study population. The majority had low-grade (67.3%), early-stage (85.1% stage I/II), endometrial cancer of endometrioid histological phenotype (74.7%). The primary treatment was surgery in 87.8% of women, 45% of whom received adjuvant therapy. LVSI and deep myometrial invasion were present in 28.9% and 36.0% respectively. During the study period, 78 women (14.7%) relapsed, 110 (20.6%) died, and the remainder were alive as at 30^th^ April 2021 (**Table 1**).

**Table 1:** Socio-demographic characteristics of the study population.

Variable	n (% total)
**Age at diagnosis**	Median age 66 years (IQR 56 73)
<65 years	243 (45.6%)
≥65 years	290 (54.4%)
**Body Mass Index (kg/m^2^)**	Median BMI 32kg/m^2^ (IQR 26, 39)
Underweight	6 (1.1%)
Normal weight	82 (15.4%)
Overweight	127 (23.8%)
Obese	318 (59.7%)
**Tumour grade**	
1	239 (44.8%)
2	120 (22.5%)
3	174 (32.7%)
**Tumour stage**	
I	397 (74.6%)
II	56 (10.5%)
III	70 (13.2%)
IV	9 (1.7%)
**Histology**	
Endometrioid	398 (74.7%)
Non-endometrioid	135 (25.3%)
**LVSI (n=530)**	
No	377 (71.1%)
Yes	153 (28.9%)
**Depth of myometrial invasion**	
<50%	341 (64.0%)
≥50%	192 (36.0%)
**Social deprivation quintile**	
Quintile I (Most deprived)	197 (37.0%)
Quintile II	125 (23.5%)
Quintile III	60 (11.3%)
Quintile IV	94 (17.6%)
Quintile V (Least deprived)	57 (10.7%)
**History of type 2 diabetes mellitus (n=535)**	
Yes	107(20.1%)
No	426(79.9%)
**Primary treatment**	
Surgery	468 (87.8%)
Hormonal (Fertility sparing reasons)	23 (4.3%)
Hormonal (Not fit for surgery)	39 (7.3%)
Radiotherapy	3 (0.7%)
**Adjuvant treatment**	
Yes	240 (45.0%)
No	293 (55.0%)
**Recurrence**	
Yes	78 (14.7%)
No	454 (85.3%)
**Survival status at end of follow up**	
Alive	423 (79.4%)
Cancer-specific mortality	76 (14.3%)
Non-cancer related mortality	34 (6.4%)
**Total**	**533 (100%)**

Bold: p < 0.05.

### Associations Between T2DM Status and Endometrial Cancer Clinico-Pathological Parameters

Women with T2DM were more obese (median BMI 36kg/m^2^) than those without (median BMI 31kg/m^2^, p<0.001). There was an association between T2DM status and socioeconomic quintile, with those from the more deprived neighbourhoods being more likely to have T2DM than those from affluent areas (p=0.045). Women with T2DM were less likely to receive hysterectomy (81.3%) compared to those without (89.4%), although the difference was not statistically significant (p=0.071). There was no evidence of an association between T2DM status and the receipt of adjuvant chemo-radiotherapy (22.5% vs 17.8%, p=0.136) or radiotherapy only (24.6% vs 18.7%, p=0.136). There was a significant correlation between T2DM status and elevated baseline CRP (p=0.013). There was no evidence of an association between T2DM status and age (p=0.141), FIGO stage (p=0.501), histological subtype (p=0.980), disease grade (p=0.654), LVSI (p=0.979) or depth of myometrial invasion (p=0.425) (**Table 2**).

**Table 2:** Baseline socio-demographic characteristics stratified by T2DM status.

Parameters	Categories	Frequency	No T2DM (n=426)	T2DM (n=107)	P value
Age (years)	<65	243	201 (47.2%)	42 (39.3%)	0.141
	≥65	290	225 (52.8%)	65 (60.7%)	
BMI (kg/m^2^)	Underweight	6	6 (1.4%)	0 (0.0%)	**0.005**
	Normal	82	74 (17.4%)	8 (7.5%)	
	Overweight	127	107 (25.1%)	20 (18.7%)	
	Obese	318	239 (56.1%)	79 (73.8%)	
FIGO stage	I	397	318 (74.6%)	79 (73.8%)	0.501
	II	56	44 (10.3%)	12 (11.2%)	
	III	70	54 (12.7%)	16 (15.0%)	
	IV	9	9 (2.1%)	0 (0.0%)	
Histology	Endometrioid	398	318 (74.6%)	80 (74.8%)	0.980
	Others	135	108 (25.4%)	27 (25.2%)	
Grade	I	239	189 (44.4%)	50 (46.7%)	0.654
	II	120	94 (22.1%)	26 (24.3%)	
	III	174	143 (33.5%)	31 (29.0%)	
LVSI (n=530)	No	377	301 (71.2%)	76 (71.0%)	0.979
	Yes	153	122 (28.8%)	31 (29.0%)	
Myometrial invasion	<50%	341	269 (63.1%)	72 (67.3%)	0.425
	≥50%	192	157 (36.9%)	35 (32.7%)	
CRP (n=355)	<5mg/dl	199	169 (59.3%)	30 (42.0%)	**0.013**
	≥5mg/dl	156	116 (40.7%)	40 (57.1%)	
Social quintile	I	197	149 (35.0%)	48 (44.9%)	**0.045**
	II	125	95 (22.3%)	30 (28.0%)	
	III	60	54 (12.7%)	6 (5.6%)	
	IV	94	81 (19.0%)	13 (12.1%)	
	V	57	47 (11.0%)	10 (9.3%)	
Primary Treatment	Surgery	468	381 (89.4%)	87 (90.7%)	0.071
	Hormonal	62	43 (10.1%)	19 (17.8%)	
	Radiotherapy	3	2 (0.5%)	1 (0.9%)	
Adjuvant therapy	None	293	225 (52.8%)	68 (63.6%)	0.136
	Chemoradiotherapy	115	96 (22.5%)	19 (17.8%)	
	Radiotherapy only	125	105 (24.6%)	20 (18.7%)	
Recurrence	No	454	370 (86.9%)	85 (79.4%)	0.054
	Yes	78	56 (13.1%)	22 (20.6%)	
Alive status	No	110	75 (17.6%)	35 (32.7%)	**0.001**
	Yes	423	351 (82.4%)	72 (67.3%)	

Bold: p < 0.05.

### Diabetic Status and Overall Survival

Women were followed up for a median duration of 40 months (range 1-165 months). The overall survival rates for the study cohort were 94% (95%CI 92-96%) at 12 months, 84% (95%CI 81-87%) at 36 months and 75% (95% CI 70-80%) at 60 months. Age at diagnosis, FIGO stage, disease grade, histology, LVSI and depth of myometrial invasion were consistent in demonstrating the expected prognostic associations. There was a 7% increase in overall mortality risk per unit increase in age (HR 1.07, 95% CI 1.05-1.09), p<0.001), but no evidence of an effect of BMI (HR 0.99, 955 ci 0.98-1.01, P=0.629). The risk of overall mortality was higher in women diagnosed with advanced-stage (FIGO III/IV) (HR 3.06, 95% CI 2.03-4.61, p<0.001), high-grade (HR 3.01, 95% CI 2.06-4.40, p<0.001), non-endometrioid (HR 2.98, 95% CI 2.04-4.34, p<0.001) endometrial cancers. LVSI and deep myometrial invasion also correlated with higher risks of death (HR 2.26, 95% CI 1.55-3.28, p<0.001 and 1.78 95%CI 1.22-2.59, P=0.003), respectively. There was a 75% increase in overall mortality for women with CRP>5.5mg/dl compared to those with CRP <5.5mg/dl (HR 1.75, 95% CI 1.09-2.80), p=0.020).

**Figure 1:**
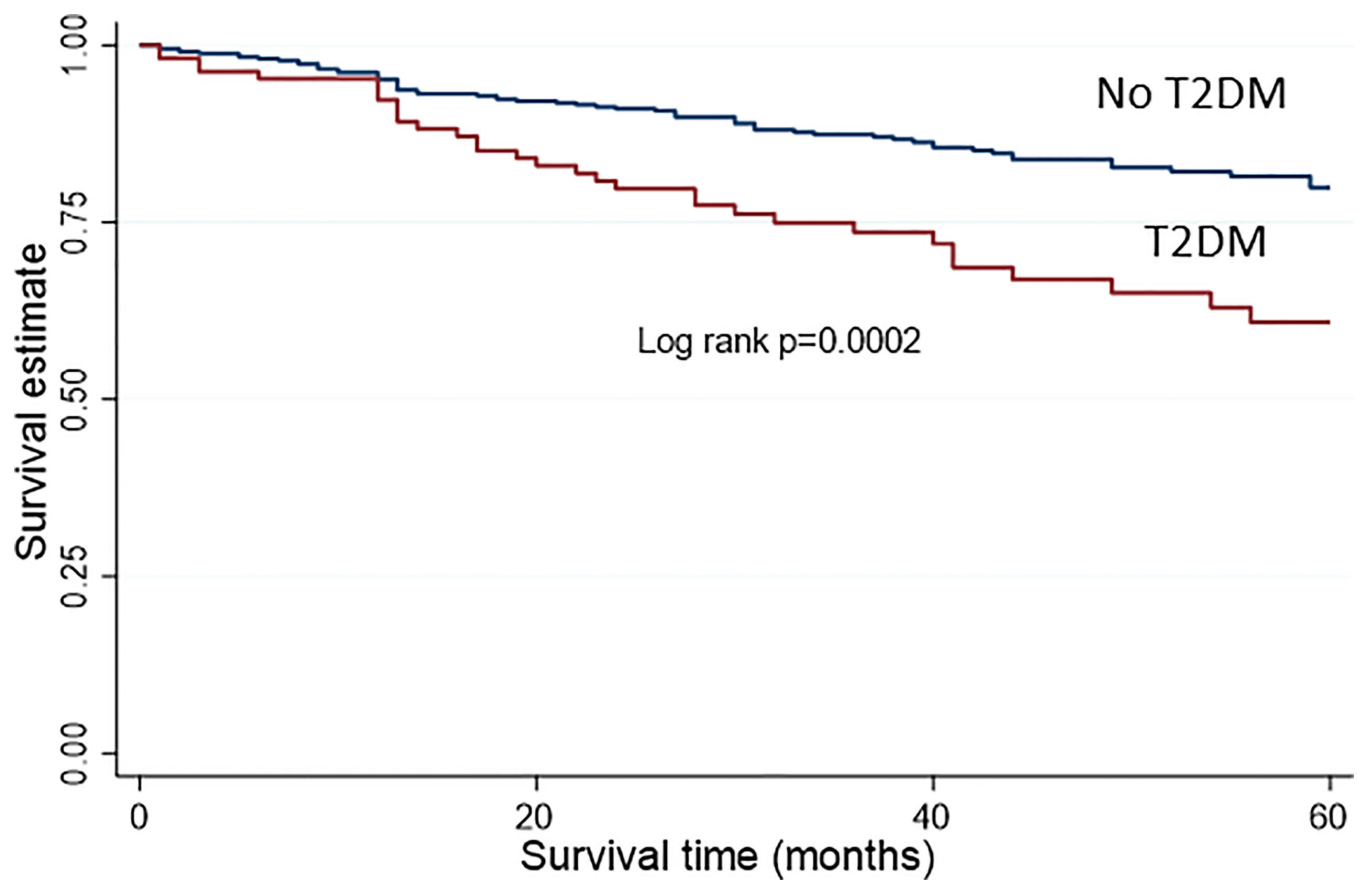
Kaplan Meier survival analysis for overall survival.

Women with T2DM had a 97% increase in overall mortality compared to those without, in univariable analysis (HR 1.97, 95%CI 1.32-2.94, p=0.001) (**Table 3** and **Figure 1**). Following adjustment for age, BMI, FIGO stage, disease grade, histology, LVSI, depth of myometrial invasion, socioeconomic quintile and baseline CRP, women with T2DM had a two-fold increase in overall mortality compared to those without (adjusted HR 2.07, 95%CI 1.21-3.55, p=0.008).

**Table 3:** Cox regression analyses of T2DM status and endometrial cancer survival outcomes with crude and adjusted hazard ratios and 95% confidence intervals.

T2DM Categories	One year survival %(95%CI)	3-year survival %(95%CI)	5-year survival %(95%CI)	Unadjusted HR (95%CI)	p-value	Adjusted HR (95%CI)	p-value
**Overall Survival**							
No T2DM	95% (92%-97%)	87% (83%-90%)	79% (74%-84%)	1.00		1.00	
T2DM	92% (85%-96%)	73% (63%-81%)	60% (47%-70%)	1.96 (1.32-2.94)	**0.001**	2.07 (1.21-3.55)	**0.008**
**Cancer-Specific Survival**							
No T2DM	96% (94%-98%)	91% (87%-93%)	84% (78%-88%)	1.00		1.00	
T2DM	95% (88%-98%)	81% (70%-88%)	71% (58%-81%)	1.73 (1.05-2.85)	**0.030**	2.15 (1.05-4.39)	**0.035**
**Recurrence free survival**							
No T2DM	94% (915-96%)	85% (815-89%)	81% (76%-86%)	1.00		1.00	
T2DM	89% (81% -94%)	72% (60%-81%)	72% (60%-81%)	1.71 (1.04-2.80)	**0.034**	2.22 (1.08-4.56)	**0.030**

Adjusted model includes age, BMI, disease histology, grade, FIGO stage, LVSI, depth of myometrial invasion, primary treatment and baseline CRP. Bold: p < 0.05.

### T2DM Status and Cancer-Specific Survival

In total, there were 110 recorded deaths, 76 (69.1%) of which were due to endometrial cancer while the remaining 34 (30.9%) were non-cancer deaths. The cancer-specific survival for the study cohort was 96% (95%CI 94-97%) at 12 months, 89% (95% CI 85-91%) at 36 months and 81% (76-85%) at 60 months. Cancer specific mortality was worse with increasing age (HR 1.06, 955 CI 1.04-1.09, p<0.001), advanced FIGO stage (HR 5.01, 95% CI 3.16-7.94, p<0.001), high-grade disease (HR 5.76, 95%CI 3.48-9.53, p<0.001), non-endometrioid histology (HR 4.84, 95%CI 3.05-7.69, p<0.001), presence of LVSI (HR 3.46, 95%CI 2.20-5.45, p<0.001), deep myometrial invasion (HR 2.23, 95%CI 1.42-3.50, p=0.001) and higher baseline CRP (HR 2.09, 95%CI 1.15-3.81, p=0.016). There was no evidence of an effect of BMI on cancer-specific deaths (HR 0.98, 95%CI 0.95-1.00, p=0.059).

Women with pre-existing T2DM had a 73% increase in cancer specific mortality compared to those without (HR 1.73, 95%CI 1.05-2.85, p=0.030) (**Table 3**). Following adjustment for age, BMI, FIGO stage, disease grade, histology, LVSI, depth of myometrial invasion and baseline CRP, those with T2DM had a two-fold increase in the risk of death from endometrial cancer compared to those without (adjusted HR 2.15, 95% CI 1.05-4.39), p=0.035).

### T2DM Status and Recurrence-Free Survival

Over the study period, there were 78 recurrences (14.7%) with a median time to recurrence 13.5 months (IQR 8-25 months). The recurrence-free survival for the study cohort was 93% (95% CI 90-95%) at 12 months, 83% (79-86%) at 36 months and 80% (75-84%) at 60 months. There was evidence of an association between recurrence free survival and age (HR 1.05, 95%CI 1.02-1.07, p<0.001), FIGO stage (HR 4.89, 95% CI 3.1-7.7, p<0.001), disease grade (HR 4.72, 95% CI 2.95-7.56, p<0.001), histology (HR 3.67, 95% CI 2.35-5.71, p<0.001), LVSI (HR 4.00, 95% CI 2.55-6.28, p<0.001), and depth of myometrial invasion (HR 2.39, 95% CI 1.53-3.73, p<0.001). There was no evidence of an effect of BMI on recurrence free survival (HR 0.99, 95%CI 0.96-1.01, p=0.255).

Women with T2DM had a 70% increase in the risk of recurrence compared to those without in univariable analysis (HR 1.71, 95% CI 1.04-2.80, p=0.034). Following adjustment for age, BMI, FIGO stage, disease grade, histology, LVSI, depth of myometrial invasion and baseline CRP, those with T2DM had a two-fold increase in the risk of disease recurrence compared to those without (adjusted HR 2.22, 95% CI 1.08-4.56, p=0.030).

## Discussion

### Main Findings

This was a prospective cohort study of 533 women with histologically confirmed endometrial cancer followed up for a median duration of 40 months. In this study, we found T2DM status to be an independent predictor of endometrial cancer outcomes. T2DM status was associated with BMI, baseline CRP and socioeconomic quintile but not FIGO stage, disease grade, histology, LVSI or depth of myometrial invasion. When these sociodemographic and clinico-pathological factors were controlled for, women with T2DM had a two-fold increase in overall mortality, cancer-specific mortality and disease recurrence. If validated in an independent cohort, T2DM status may help refine endometrial cancer risk assessment and when considered alongside other clinico-pathological parameters, may guide decisions about adjuvant therapy in endometrial cancer.

### Strengths and Limitations

This study benefits from a large sample size of women with endometrial cancer recruited to several population-based studies that posed few restrictions according to clinico-pathological parameters, alleviating concerns about the possibility of selection bias. The availability of data on socio-demographic and clinico-pathological characteristics allowed for a robust adjustment for confounding factors and effect modifications. To our knowledge, this is the first study to adjust for baseline CRP, a parameter that is known to be associated with T2DM status and which has been reported to independently predict outcomes in endometrial cancer (6). The established endometrial cancer prognostic factors, including FIGO stage, disease grade, histological subtype, LVSI and depth of myometrial invasion, were consistent in demonstrating the expected associations. We did not collect comorbidity or medication use data, and neither did we have information regarding endometrial cancer molecular subgroup for our study cohort, and this may have led to an over- or under-estimation of endometrial cancer outcomes. The generally favourable prognosis of endometrial cancer and consequent low event rate affects the reliability of our conclusions. The relatively small number of women with T2DM reduces the precision of our estimates. Finally, as this was a prospective study of mostly White British women managed at a specialist cancer centre, we cannot necessarily generalise our study findings to women from other treatment centres, nationalities or ethnicities.

### Interpretation

Large epidemiological and mechanistic studies have been consistent in suggesting an association between T2DM and endometrial carcinogenesis (13, 16, 17, 19, 20). Women with T2DM are at a 62% increased risk of endometrial cancer, independent of obesity, compared to those without (13). Insulin resistance, hormonal imbalance and systemic inflammation are the three main biological pathways implicated in endometrial cancer development (14). Insulin resistance results in hyperinsulinemia and hyperglycaemia which alongside chronic inflammation promotes endometrial tumorigenesis and metastasis by the direct pro-proliferative and anti-apoptotic effect of insulin and insulin growth factor (IGF-1) on endometrial cells (15). However, whether T2DM independently impacts on endometrial cancer outcomes is unclear. The systematic review of relevant cohort studies by Liao and colleagues concluded that the evidence for an association between T2DM and endometrial-cancer specific mortality was low quality (17). Of the six included studies, only two reported relative risk ratios and were quantitatively synthesized (summary estimate RR 1.32 [1.10-1.60]) (17). One study reported a hazard ratio of 1.64 that was not statistically significant (21) while the remaining three studies (22–24) reported standardised mortality ratios that could not be pooled together. In our study, we show evidence that T2DM impacts on endometrial cancer overall, cancer-specific and recurrence free survival, following robust adjustment for important clinico-pathological confounders. T2DM status was associated with BMI, socioeconomic quintile and baseline CRP, consistent with previous work (25–27). Our findings are consistent with the recent report by Nagle and colleagues of a two-fold increase in cancer-specific mortality in endometrial cancer patients with T2DM compared to those without (28). If validated in a larger independent cohort, our findings have important clinical and therapeutic implications. Pre-existing T2DM was recorded for 20% of patients, for whom personalised care and careful follow-up is justified.

The impact of T2DM on endometrial cancer outcomes may be related to tumour (cancer stage, disease grade, and tumour biology), patient (age, obesity, and other comorbidities) or health care factors (variation in type of care offered) (29). T2DM can impact on the FIGO stage at endometrial cancer diagnosis. It is indeed plausible that having a comorbidity like T2DM can result in increased contact with the National Health Service, thus creating opportunities for the early diagnosis of endometrial cancer. Conversely, pre-existing T2DM may distract either, or both, the patient and health care providers, resulting in delayed cancer diagnosis and poor outcomes (29). In our study, we found no evidence of an association between T2DM status and FIGO stage, and there was minimal evidence of confounding by FIGO stage; correction for FIGO stage did not considerably affect the T2DM hazard ratios. Comorbid diabetes may also influence disease grade and tumour biology. Mechanistically, the pro-proliferative and anti-apoptotic effect of insulin and IGF on endometrial cells, induced by insulin resistance in T2DM may be expected to lead to more aggressive endometrial cancer phenotypes (15, 30). In our study, however, there was no evidence of an association between T2DM status and disease grade or histological subtype; and neither mediated the link between T2DM and endometrial cancer outcomes, as the hazard ratios remained significant after adjusting for these variables. T2DM may also affect endometrial cancer outcomes through patient related factors such as age, BMI and the presence of related comorbidities. However, both age and BMI were adjusted for in the multivariable analyses, suggesting that they could not have underpinned our study findings.

Healthcare and treatment related factors may also explain the association between T2DM and endometrial cancer outcomes (29). There is evidence to suggest that cancer patients with a comorbidity are less likely to be offered curative treatment than those with no comorbidity (31). Indeed, women with T2DM are more likely to have other comorbid conditions such as hypertension and heart disease and thus may be less likely to be offered surgery, compared to those without (31, 32). Furthermore, women with T2DM who undergo surgery may be at an increased risk of peri-and post-operative complications that contribute to poor outcomes (31, 33). Women with comorbidities like T2DM may also be less likely to receive adjuvant chemotherapy, be more liable to receive a reduced dose and more likely to not complete treatment (34–38). In our study, there was no evidence of a significant difference in treatment allocation by T2DM status in either the primary or adjuvant settings. However, treatment-related factors relating to dosing and completion of treatment cannot be ruled out. Furthermore, limited data suggest that metformin is associated with improved overall and progression-free survival outcomes in endometrial cancer (39, 40). Two meta-analyses, involving 1,594 and 3,923 women with endometrial cancer respectively, concluded that metformin reduces the risk of recurrence and death in endometrial cancer survivors (39, 40). However, we were unable to include this in our multivariable model due to lack of data on medication use in our cohort.

In conclusion, we found that T2DM confers an increased risk of death from endometrial cancer. Well-designed longitudinal studies with large sample sizes are now needed to confirm these findings.

## Data Availability Statement

The original contributions presented in the study are included in the article/supplementary material. Further inquiries can be directed to the corresponding author.

## Ethics Statement

The primary research studies were: Metformin (North West Research Ethics Committee, NW REC, 11/NW/0442, approved 19 August 2011), Weight loss (NW REC, 12/NW/0050, approved 23 January 2012), PREMIUM (NW REC, 14/NW/1236, approved 23 September 2014), PETALS (NRES Committee North West, Lancaster, 15/NW/0733, approved 18 September 2015) and DETECT (NW REC, Greater Manchester, 16/NW/0660, approved 16 September 2016). The patients/participants provided their written informed consent to participate in this study.

## Author Contributions

Supervision and funding acquisition, EJC; Conceptualization, KN and EJC; study design, KN and EJC; statistical analysis, KN; writing, original draft preparation, KN, Review and editing KN, HJA, and EJC; all authors provided critical comments, read, and approved the final version for publication.

## Funding

KN is supported by a Cancer Research UK (CRUK) Manchester Cancer Research Centre Clinical Research Fellowship (C147/A25254) and the Wellcome Trust Manchester Translational Informatics Training Scheme. EJC is supported by the NIHR Manchester Biomedical Research Centre (IS-BRC-1215-20007) and an NIHR Advanced Fellowship (NIHR300650). HJA is supported by a Manchester University NHS Foundation Trust Clinical Research Fellowship.

## Conflict of Interest

The authors declare that the research was conducted in the absence of any commercial or financial relationships that could be construed as a potential conflict of interest.

## Publisher’s Note

All claims expressed in this article are solely those of the authors and do not necessarily represent those of their affiliated organizations, or those of the publisher, the editors and the reviewers. Any product that may be evaluated in this article, or claim that may be made by its manufacturer, is not guaranteed or endorsed by the publisher.
